# Impact of war and forced displacement on children’s mental health—multilevel, needs-oriented, and trauma-informed approaches

**DOI:** 10.1007/s00787-022-01974-z

**Published:** 2022-03-14

**Authors:** David Bürgin, Dimitris Anagnostopoulos, the Board and Policy Division of ESCAP, Dimitris Anagnostopoulos, Maeve Doyle, Stephan Eliez, Jörg Fegert, Joaquin Fuentes, Johannes Hebebrand, Manon Hillegers, Andreas Karwautz, Eniko Kiss, Konstantinos Kotsis, Milica Pejovic-Milovancevic, Anne Marie Räberg Christensen, Jean-Philippe Raynaud, Sofie Crommen, Füsun Çuhadaroğlu Çetin, Vlatka Marsanic Boricevic, Laura Kehoe, Maja Drobnič Radobuljac, Renate Schepker, Robert Vermeiren, Звepeвa Haтaлья, Benedetto Vitiello, Thorsten Sukale, Marc Schmid, Jörg M. Fegert

**Affiliations:** 1 Department of Child and Adolescent Psychiatry/Psychotherapy, University Hospital Ulm, University of Ulm, Steinhövelstrasse 5, 89073 Ulm, Germany; 2 Child and Adolescent Psychiatric Research Department, Psychiatric University Hospitals, University of Basel, Basel, Switzerland; 3 National and Kapodistrian, University of Athens, Athens, Greece; 4 Division of Child Neurology and Psychiatry, Department of Public Health and Pediatric Sciences, Regina Margherita Pediatric Hospital, University of Turin, Turin, Italy

**Keywords:** Children, War, Refugee, Forced migration, Mental health, Burden, Human rights, Children’s rights, Psychopathology, Post-traumatic stress disorder, Depression, Anxiety, Trauma

## Abstract

The infliction of war and military aggression upon children must be considered a violation of their basic human rights and can have a persistent impact on their physical and mental health and well-being, with long-term consequences for their development. Given the recent events in Ukraine with millions on the flight, this scoping policy editorial aims to help guide mental health support for young victims of war through an overview of the direct and indirect burden of war on child mental health. We highlight multilevel, need-oriented, and trauma-informed approaches to regaining and sustaining outer and inner security after exposure to the trauma of war. The impact of war on children is tremendous and pervasive, with multiple implications, including immediate stress-responses, increased risk for specific mental disorders, distress from forced separation from parents, and fear for personal and family’s safety. Thus, the experiences that children have to endure during and as consequence of war are in harsh contrast to their developmental needs and their right to grow up in a physically and emotionally safe and predictable environment. Mental health and psychosocial interventions for war-affected children should be multileveled, specifically targeted towards the child’s needs, trauma-informed, and strength- and resilience-oriented. Immediate supportive interventions should focus on providing basic physical and emotional resources and care to children to help them regain both external safety and inner security. Screening and assessment of the child’s mental health burden and resources are indicated to inform targeted interventions. A growing body of research demonstrates the efficacy and effectiveness of evidence-based interventions, from lower-threshold and short-term group-based interventions to individualized evidence-based psychotherapy. Obviously, supporting children also entails enabling and supporting parents in the care for their children, as well as providing post-migration infrastructures and social environments that foster mental health. Health systems in Europe should undertake a concerted effort to meet the increased mental health needs of refugee children directly exposed and traumatized by the recent war in Ukraine as well as to those indirectly affected by these events. The current crisis necessitates political action and collective engagement, together with guidelines by mental health professionals on how to reduce harm in children either directly or indirectly exposed to war and its consequences.

## Background and introduction

“Children are both our reason to eliminate the worst aspects of armed conflict and our best hope of succeeding in that charge.”—Dame Graça Machel, human rights activist, former first lady of Mozambique and South Africa (1996).

The “United Nations Convention on the Rights of the Child” (UNCRC), ratified by almost all nations of the world, states the fundamental rights of children and especially the right to life, health, and development, bans discrimination, and calls for mandates the protection of children’s interests [1, 2]. In a later resolution, the UN Security Council, adopted the UNCRC with an Optional Protocol to the UNCRC about the “Rights of the Child on the Involvement of Children in Armed Conflicts”, and determined that violence against children in armed conflicts poses a threat to durable peace, security, and development [3, 4]. The infliction of war and military aggression upon children can thus be considered a violation of children’s basic human rights. From a developmental and psychopathological perspective, it can result in persistent impairment in health, well-being, and developmental potential.

Exposure to war, living in conflict zones, flight, and forced migration may create or increase the risk for broad sequalae of direct and indirect risks for physical and mental health, more so for children and their caregivers, and deprives children from developmental opportunities and basic resources. Effects on children’s health result from actual violence against themselves and their families and from inadequate health care, malnutrition, infectious diseases, and distress caused to their families [5–8]. Regarding maternal and child health, studies show a trend towards worse pregnancy-related outcomes, such as a higher rate of preterm births among refugee mothers, higher rates of stillbirths, children with low-birth weight, and increases and prenatal and postnatal mortality [5, 9, 10]. The global burden of mental health consequences of war and migration are enormous with high prevalence rates of depression and post-traumatic stress disorder (PTSD) in war affected countries [11–13]. Flight and forced migration are further risk factors for children’s mental health, even more so for unaccompanied minors separated from their parents [10]. Next to consequences for physical and mental health, armed conflicts inflict high broad costs as basic social services deteriorate, existing communal divisions enlarge, local economies collapse, and schooling is disrupted, and educational opportunities decrease [14, 15]. Taken together, the risks inflicted by war, living in conflict zones, flight, and forced migration upon children are many fold and might have lifelong impacts on physical, mental, social well-being, and development.

Within UNICEF’s recent report on “The State of the World’s Children 2021”, the current COVID-pandemic is considered the tip of the mental health iceberg—which has been ignored for too long [16]. The mental health burden being inflicted on Europe’s children by the recent war in Ukraine is the part of the iceberg with the potential to sink the ship. Besides the children directly hit by the war, all other children across Europe might also be indirectly affected as the media carry the war into every family home. This adds another layer of insecurity and anxiety on children who are already burdened from the COVID-pandemic that has been accompanied by higher levels of anxiety and a reduced quality of life [17, 18].

Thus, the current crisis calls for political action and collective engagement to prevent and reduce the harm to children while supporting all those involved in their care. The aim of this scoping policy editorial is help guide mental health support for young victims of war through an overview of the direct and indirect burden of war on child mental health and of multilevel, need-oriented, and trauma-informed approaches to regaining and sustaining outer and inner security after exposure to the trauma of war.

## Methods

Due to the complexity and urgency of the research objective and the aim to highlight timely guidance for caregivers and first responders considering the recent war-outbreak in Ukraine, we searched for studies that assessed and addressed the impact of war and flight on children’s mental health in major conflict and war zones. A selective review of the literature was performed using multiple search strategies. A literature search of relevant publications through the 1st of March 2022 was conducted using the PubMed and Google Scholar databases. The following key words were used in varying combinations: ‘children’, ‘war’, ‘flight’, ‘trauma’, ‘trauma-informed’, and ‘mental health’. For this review, childhood was defined as being under the age of 18 according to the UNCRC [2] and “children” refers to this age range if not otherwise explicitly specified. Reference lists from relevant reviews were examined for possible additional studies. Studies were assessed qualitatively.

## Impact of war and forced displacement on children’s mental health

### Immediate psychological distress and stress reactions in children

“It does not take much imagination to think of the experiences children may have had in fleeing from their homes under threat, witnessing fighting and destruction, seeing violent acts directed at their loved ones, leaving their friends and possessions behind, marching or being transported in crowded vehicles, spending months in transit camps, and eventually finding temporary respite in a country at peace while the authorities decide whether the family can be granted permission to remain legally and indefinitely.”—Yule [8, p. 696].

Children exposed to war and flight show a broad range of possible distress and stress reactions e.g. specific fears, dependent behavior, prolonged crying, lack of interest in the environment, and psychosomatic symptoms, as well as aggressive behaviors [19–23]. Children’s play can also be affected, for instance with the emergence of morbid themes, restriction in fantasy play, and social withdrawal [23–25]. It is important to appreciate that it is not merely the ‘objective’ nature of the specific experience that is important, but how each child subjectively perceives, appraises, and interprets that experience [8]. Thus, there can be huge differences in children’s stress reactions to what may seem from the outside to be similar experiences [8]. Also, it is important to consider that children respond differently to the stress of violent exposures depending on their developmental level, and that it is necessary to understand such stress reactions within the context of their social-emotional and cognitive development [26]. Taken together, the stress reactions of children comprise a broad array of potential emotional and behavioral reactions to different distressing experiences that depend not only on the objective nature of the experience but on the subjective perception by the child.

### Post-traumatic stress, anxiety, and depressive disorders

Next to immediate stress reactions in children, studies show a higher prevalence of certain mental disorders among children during and post-conflict as compared with the general population [27]. Most studies have focused mainly on PTSD as the primary outcome, whereas others also assessed depression and anxiety disorders [27]. In general, multiple meta-analyses document a high burden of mental disorders and psychopathology on conflict-affected, internally displaced, and refugee populations [28–33]. A meta-analysis of eight studies of child and adolescent refugees and asylum seekers reported a 22.7% prevalence of PTSD, 13.8% of depression, and 15.8% of anxiety disorders [29, 30]. These data raise concern, as studies estimate the absolute number of war-exposed children between 1989 and 2015 to be around 400 million [11, 12].

The high psychological burden on refugees underscores the need for continuous mental health care over and beyond the initial period of resettlement [29, 30]. Some studies show the prevalence for mental disorders in the first years of resettlement, only clearly to be increased for PTSD, however 5 years after resettlement the rates for depressive and anxiety disorders are also found to be increased [34]. A meta-analysis on risk factors for PTSD in children shows pre-trauma factors and objective measures associated with the event itself to only generate small to medium effects, whereas medium to large effect sizes were found for many factors associated-with subjective experience of the event and post-trauma variables, such as low social support, perceived life threat, social withdrawal, poor family functioning, and thought suppression [35]. These findings indicate that peri-traumatic factors and post-event factors have an important role in the development of PTSD in children [35]. Taken together, these reports point to the urgent need for support during and after war-related exposures, as well as for long-term mental health care for young people and families fleeing war and seeking refuge.

### Broad sequalae: separation from parents, loss of outer and inner safety

During war children often become separated from one or both parents as seen in recent pictures of children fleeing with their mothers at the Ukrainian border and leaving their fathers behind. Research on attachment has shown the damaging consequences of deprivation and separation from parents in a variety of contexts and circumstances [36, 37]. Overall, research has found that parent–child separation has consistently negative effects on children’s social-emotional development, well-being, and mental health [38]. Another major problem during flight from danger is the loss of safe places where to live with consequent high levels of prolonged stress. Within social safety theory, cognitive schemas of social safety are thought to develop during childhood and adolescence in relation to a child’s appraisal of themself, the social world, and the projected future [39]. Such perceptions are shaped by the actual situations that the child encounters (for instance, exposure abuse and violence) and by the meaning and narratives that people in general, and their parents in particular, attribute to such events [39]. Thus, children exposed to war are ripped of their safety at multiple levels, from the individual feeling of being safe during separation and loss of family life, to being away from friends, their homes, cities, and at times even their countries.

Taken together the impact of war on children is tremendous, ranging from immediate stress-responses and increased risk for specific mental disorders—PTSD, depression, and anxiety—to the broad consequences of separation from parents and the loss of safety. Thus, children’s experiences during and directly after war are in marked contrast with need and right to develop in a safe, secure, and predictable environment [8].

## Multilevel, needs-oriented, and trauma-informed approaches aiming to reduce the impact of war on children’s mental health

**Fig. 1:**
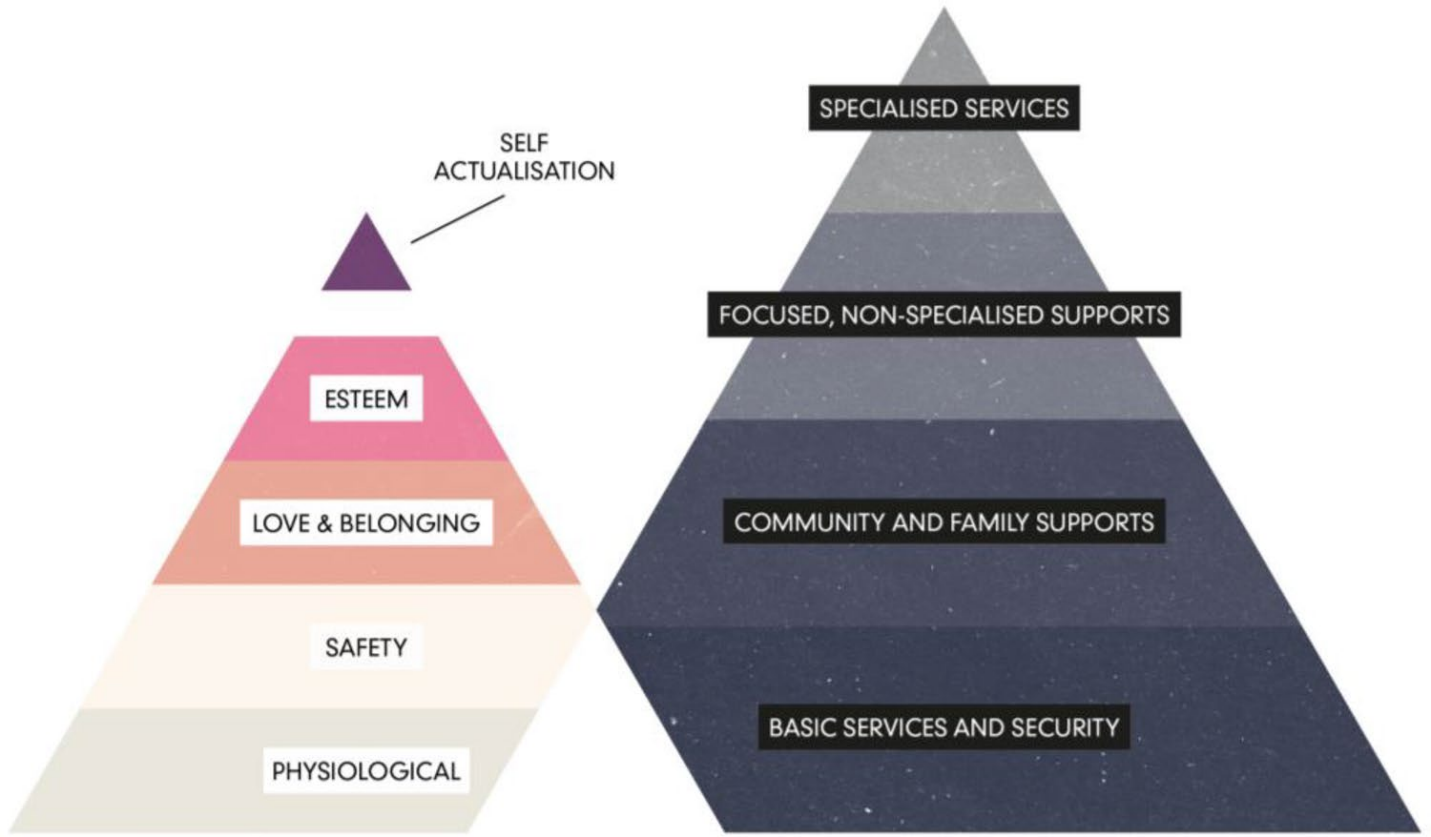
Left pyramid: hierarchy of (children’s) basic needs [45, 46]. Right pyramid: multilevel intervention pyramid for mental health and psychosocial support in emergencies; within this pyramid all layers of the pyramid are important and should ideally be implemented concurrently [42]

Interventions supporting war-affected children should be comprehensive, sustainable, and devoid of harm [40]. Support should be multilevel, resilience-oriented, multidisciplinary, and tailored to the needs of subgroups and individuals [40, 41]. The multilevel intervention pyramid for mental health and psychosocial support in emergencies proposed by the Inter-Agency Standing Committee (IASC) [42] includes four levels of intervention: provision of basic services and security, community and family support, focused non-specialized support, and specialized supports (see Fig. 1, right). In such a multilevel understanding of intervention, all layers of the pyramid are important and should ideally be implemented concurrently according to the need of the individual. Such a multilevel understanding of intervention is closely linked to a socioecological and multisystemic understanding of resilience, where resilience is linked to an individual’s ability to harness resources, but also to the resources being provided to be harnessed [43, 44]. Thus, multisystemic and multilevel approaches are required.

Experiences that children in war and those searching for refuge face are in utter contrast to what can be consider to be the basic needs of every child: basic physiological and safety needs, including the need for shelter and food; the need for safety and security; the need for continuity of care by a loved one; and the need for good schooling and opportunities to develop and thrive [8, 46]. Basic human needs were described by Maslow [45] in his hierarchy of basic needs visualized as a pyramid (see Fig. 1, left) and discussed in the context of child development [46, 47]. These and other basic needs represents also recognized rights, including the right to be protected during war and the right to receive help if affected by war to regain health and dignity (Article 38 & 39, [1, 2]). Whether living in a warzone, being on the flight, or staying in a new country that offers refuge, children’s needs should be properly assessed and accordingly met, from the more basic physiological needs and moving up along the pyramid.

Multilevel help efforts and interventions should be trauma-informed and, as such, “grounded in an understanding of and responsiveness to the impact of trauma, that emphasizes physical, psychological, and emotional safety for both providers and survivors, and that creates opportunities for survivors to rebuild a sense of control, self-efficacy and empowerment” [48, p. 82]. Trauma informed care is built around three important pillars: safety, connections, and managing emotions [49]. It, thus, tries to translate trauma research into practice to inform and improve care efforts, practically address trauma, and promote resilience thus improving outcomes [50].

Taken together, mental health and psychosocial support and intervention for war affected children should be multilevel, targeted towards the child’s needs, from basic physical needs upwards, and should be trauma-informed and thus strength- and resilience-oriented.

### Multilevel interventions for children exposed to war and forced migration

#### (a) Provide immediate aid and intervention

Immediate humanitarian aid needs to be targeted towards addressing children’s basic physiological and outer safety needs and must ensure children’s access to basic services and safety from direct harm (lower levels of needs and intervention pyramids in Fig. 1). These basic services include the provision of food, shelter, water, and basic health care, established in participatory, safe, and socially appropriate ways [42]. Examining interventions for war affected children underlines “the importance of providing children with safety and a sense of security, as well as addressing basic needs and establishing trust with the child” [51]. Such non-specific interventions have the goal to reduce stress, to provide safe areas and shelters; to restore or reactivate protective factors [52]. Building up and adding to basic services, psychological first aid should aim at reducing initial post-trauma distress and supporting adaptive functioning [53]. Psychological first aid consists of eight core actions that address: contact and engagement, safety and comfort, stabilization, information gathering, practical assistance, connection with social supports, information on coping support, and linkage with collaborative services [53, 54]. From a trauma-informed perspective, it is important to build back some normal in the abnormal. This might include to rebuild daily structures and routines, to provide safe places for children to play and interact with others, to provide relationship offers to children and thus the opportunity to talk about emotions, to be aware of oneself and others, and to have the opportunity to regulate one’s own emotions with a caregiver.

#### (b) Assess and screen for mental health burden and needs

When guiding help efforts, it is important to acknowledge that some children might ‘only’ require a sense of safety and support by family and close others, whereas others might be in need of more complex and focused psychosocial support that addresses the different stress reactions and emotional and behavioral problems that manifest [8]. Thus, a phased model of intervention with a stepped care approach is needed [42, 55, 56]. For such an approach the assessment of children’s needs and thus a screening for their mental health burden is indicated [55]. The detection through proper assessment and the subsequent treatment of mental health issues among refugee children should be a priority to reduce war-associated morbidity [57]. Assessing the needs of younger children can be especially difficult [23, 57, 58]. Using validated instruments to assess the circumstances of refugee minors that can be easily and widely implementable, such as in online-based screening instruments, is of great importance (as for instance implemented within “PORTA”) [59–61]. An adequate assessment and screening of children’s mental health burden and needs, and their resources is a precondition for indicated and targeted intervention.

#### (c) Provide evidence-based interventions for groups and individuals

Based on an individual assessment of the burden and needs of children, appropriate, effective, and efficacious evidence-based treatments should be made available. Different forms of evidence-based programs and treatments exist, ranging from lower-threshold group-based interventions implemented by non-specialists to individual evidence-based trauma-focused psychotherapy by trained professionals [62–65]. With a stepped care approach in mind, interventions with lower participation thresholds and fewer sessions can be implemented in group-based settings to reduce the mental health burden of minors—e.g., as implemented in the program “Mein Weg” (English “My Way”) [20, 21, 66]. Another short-term low-threshold program is START **(**Stress-Trauma symptoms-Arousal-Regulation-Treatment), for which there is preliminary evidence of efficacy in improving emotion regulation in adolescents with traumatic exposures and which has been recently adapted and tested in adolescent refugees [67–69]. In a meta-analyses of 36 studies, different treatments were found to have overall large effect sizes, with evidence-based classroom-based interventions showing effect sizes of equal magnitude compared other individual therapies [62, 63]. In a review of 25 evidence-based interventions for children and youth affected by armed conflict, several practice elements were seen in more than 50% of the intervention protocols [62]. These elements included: access promotion, psychoeducation for children and parents, insight building, rapport building techniques, cognitive strategies, use of narratives, exposure techniques, and relapse prevention [62]. Taken together, a growing body of research demonstrates the efficacy and effectiveness of evidence-based interventions from low-threshold and short-term group-based interventions to long-term individualized and evidence-based psychotherapy. Such interventions, if indicated and accessible, should be made available.

#### (d) Provide appropriate post-migration infrastructures and social environments that foster mental health

For long-term help, it is important to consider providing appropriate physical infrastructures where people seeking refuge can regain and sustain their mental health. Worse mental health outcomes are seen in refugees living in institutional accommodation with restricted economic opportunities. Humanitarian aid that improves living conditions is likely to have a positive impact on mental health as well [33]. Also, factors after migration may moderate the ability of those seeking refuge to recover from pre-migration trauma, stressing the importance to address post-migration stressors to improve mental health [70]. With a trauma-informed perspective, infrastructures and social environments are needed that provide children with the opportunity to develop in a safe environment that is child appropriate regarding social interaction, education, and strength promotion. Taken together, post migration structures are needed that suit the ‘higher’ needs of children over and beyond providing initial safety. This includes social interaction and a sense of belonging, education, and a promotion of individual strengths.

#### (e) Support parents during and after war

Supporting children does also mean to support parents and enable them to care for their children. Studies have shown that more war-exposed parents show less warmth and more harshness toward their children, which partly explains child adjustment [71]. Overall, studies have consistently documented the protective nature of parental support on the mental health of children in armed conflict [72]. In preschool children, studies show the emotional sensitivity and regulation, attachment style, and PTSD symptoms of mothers as central moderators between traumatic exposures and mental health consequences of their children [73]. Such findings also emerged between parental and children’s psychopathology [23]. Another moderator of the exposure-outcome association for children is the family environment and parental functioning [23]. In general, interventions in war zones should ensure the least disruption to communities and families and should wherever possible involve parents in preventive or treatment programmes for children [74]. Thus, promoting healthy development of war-affected children should also focus on supporting their parents. In particular, helping parents to maintain warm interactions despite war atrocities, thus limiting harshness as much as possible, might foster healthy child adjustment [71].

#### (f) Support indirectly affected children

War does not only affect those children directly exposed, but also those indirectly impacted through mass media bringing the pictures of war into their homes. Children cannot protect themselves against the themes addressed in the news and omnipresent pictures of war on television, in newspapers and on social media. Perceived anxiety of war in parents might lead to a loss of sense of security and enhanced anxiety in children. As discussed above, cognitive schemas of social safety are developed during childhood and adolescence in relation to a child’s appraisal of themselves and the social world around and by the meaning and narratives that people in general and their parents in particular attribute to such events [39]. Children indirectly affected by war, gladly and mostly are not separated from their families, and do not have to leave their friends, home, and city behind, but they can experience this social threat on the collective level—hearing about missiles and nuclear deterrence. Considering the COVID-pandemic and its burden onto families with already high levels of anxiety and mental health problems and a reduced quality of life of children and their parents [17, 18, 75–79]—the current feeling of unsafety has the potential to create a double jeopardy. Vulnerable children and adolescents must thus cope with both war in Europe and the consequences of a two-year pandemic. This situation will likely create further challenges and struggles for already challenged child and adolescent psychiatric service providers dealing with the increased post-COVID mental health needs.

*How can parents talk to their children?* It is important to find a balance between telling the truth about the adversities of war and to still convey hope and signs of physical as well as emotional safety. It is important to protect children from constant inundation of overwhelming information about the current situation especially from disturbing images in the media and to at times turn off one’s own devices. Providing relationship opportunities and encourage children to talk about their feelings and to share one’s own feelings can be helpful. Much can also be done through simple, concrete acts to support children and families directly hit by the war, such as donations of money or clothes. When children see that parents do something for refugee children, this might help to connect their stress into an essential context of meaning and action.

## Implications for European Child and Adolescent Psychiatry

Considering the direct or indirect impact of war on children’s mental health: what can the European Child and Adolescent Psychiatric Services and in particular the European Society for Child and Adolescent Psychiatry (ESCAP) contribute?

The current war outbreak in Ukraine and the high mental health burden of children following the COVID pandemic underline the need to focus on mental health and adequate help and intervention for those in need. We should support our colleagues in Ukraine, Russia, and the countries where people are finding refuge, e.g., by providing help to colleagues and emergency personnel. We will continue to advocate for the increased mental health needs of children in the actual situation. With the hope that European nations provide new funds and resources to address the mental health crises of children and to launch large-scale programs to counteract these challenges. Longitudinal and developmentally oriented follow up is needed as early and chronic traumatic stress often does not heal naturally in those strongly affected [80–82]. Children's mental health has been challenged due to recent crises and adversities, as discussed, the COVID-pandemic and war in Ukraine creating a recent double jeopardy. These however can be seen as current episodes of a continuum of crises within Europe, at its borders, and worldwide, which constitute to a cumulative negative impact on children's mental health, well-being, and development—to mention two: the ongoing refugee crises around the Mediterranean and future implications of climate change on children’s mental health [76]. The actual crisis also shows that children in our own European spheres are affected by war, but that we need be aware of the much larger number of war-affected families worldwide [13].To address this need, the European Union and other nations need to invest into the future of the world’s children, as they are, in the words of Dame Graça Machel (1996), “both our reason to eliminate the worst aspects of armed conflict and our best hope of succeeding in that charge”. Thus, it’s time to come together and give our absolute best to protect and support our children!
